# Tree species hyperdominance and rarity in the South American Cerrado

**DOI:** 10.1038/s42003-025-07623-w

**Published:** 2025-05-03

**Authors:** Facundo Alvarez, Ben Hur Marimon-Junior, Beatriz S. Marimon, Hans ter Steege, Oliver L. Phillips, Renata Dias Françoso Brandão, Eraldo A. Trondoli Matricardi, José Roberto Rodrigues Pinto, Frederico Augusto Guimarães Guilherme, Marcelo Leandro Bueno, Sabrina Miranda, Bruno Machado Teles Walter, Cássia B. Rodrigues Munhoz, Edson de Souza Lima, Fabiana de Góis Aquino, Henrique Augusto Mews, José Felipe Ribeiro, Maria Antônia Carniello, Mercedes Maria da Cunha Bustamante, Ricardo Haidar, Paulo Sérgio Morandi, Edmar Almeida de Oliveira, Zenésio Finger, Eder Carvalho das Neves, Fernando Elias, Immaculada Oliveras Menor, Ana Lyz Machado Parreira, Eddie Lenza de Oliveira, Eduardo Queiróz Marques, Reginal Exavier, Carla Heloísa Luz de Oliveira, Nayane Cristina Candida dos Santos Prestes, Simone Matias de Almeida Reis, Wesley Jonatar Alves da Cruz, Ted R. Feldpausch

**Affiliations:** 1 Universidade do Estado de Mato Grosso; Campus de Nova Xavantina, PPG Ecologia e Conservação, Nova Xavantina, Mato Grosso, Brazil; 2 Naturalis Biodiversity Center, Leiden, The Netherlands; 3 Utrecht University; Quantitative Biodiversity Dynamics, Dept. of Biology, Utrecht, The Netherlands; 4 University of Leeds; School of Geography, Leeds, UK; 5 Universidade Federal de Lavras; Departamento de Ciências Florestais, Lavras-MG, Brazil; 6 Universidade de Brasília; Departamento de Engenharia Florestal, Brasília-DF, Brazil; 7 Universidade Federal de Jataí; Instituto de Biociências, Jataí, Goiás Brazil; 8 Universidade do Estado de Mato Grosso do Sul; Laboratório de Macroecologia e Evolução, Mundo Novo, Mato Grosso do Sul Brazil; 9 Universidade Estadual de Goiás; Unidade Universitária de Palmeiras de Goiás, Goiás, Brazil; 10 Embrapa Recursos Genéticos e Biotecnologia; CEN Herbarium, Brasília, Brazil; 11 Universidade de Brasília; Instituto de Ciências Biológicas, Departamento de Botânica, Brasília-DF, Brazil; 12 Embrapa Cerrados, Brasília-DF, Brazil; 13 Universidade Federal de Rondonópolis; Instituto de Ciências Exatas e Naturais, Rondonópolis, Mato Grosso Brazil; 14 Universidade do Estado de Mato Grosso; Campus de Cáceres, Cáceres, Mato Grosso Brazil; 15 Universidade de Brasília; Departamento de Ecologia, Brasília, Brazil; 16 Universidade Federal do Tocantins; Departamento de Engenharia Ambiental, Palmas, Tocantins Brazil; 17 Universidade Federal de Mato Grosso; Faculdade Engenharia Florestal, Cuiabá, Mato Grosso Brazil; 18 Universidade Federal Rural da Amazônia, Capitão Poço, Pará, Brazil; 19 AMAP, University of Montpellier, CIRAD, IRD, CNRS, INRAE, Montpellier, France; 20 Environmental Change Institute, School of Geography and the Environment, University of Oxford, Oxford, UK; 21 Universidade de São Paulo, São Paulo, Brazil; 22 Universidade Federal do Acre; Centro de Ciências Biológicas e da Natureza, Rio Branco, Acre Brazil; 23 University of Exeter; Geography, Faculty of Environment, Science and Economy, Exeter, UK

**Keywords:** Biodiversity, Plant ecology, Biogeography

## Abstract

The South American Cerrado, the largest savanna of the Americas and the world's most tree-biodiverse, is critically endangered, with just 8% protected and more than half deforested. However, the extent of its tree diversity and abundance remains poorly quantified. Using a unique biome-wide eco-floristic dataset with 222 one-hectare plots, we estimate the Cerrado has ~1605 tree species and has extreme hyperdominance, with fewer than 2% (30 species) accounting for half of all trees. A single family, Vochysiaceae, represents 17% of all trees, and the most abundant species, Qualea parviflora, accounts for 1 in 14 trees. In contrast, 63% of the species are rare, with fewer than 100 trees across all plots. Remote sensing and spatial modelling suggest the Cerrado has lost 24 billion trees since 1985, equivalent to three times the Earth's human population. We estimate up to 800 tree species may remain undetected in Cerrado ecosystems and could face extinction in a few decades due to deforestation. This hyperdominance parallels patterns in Amazonian forests and highlights risks both biomes face for species loss due to fragmentation, deforestation, and land-use change. Our findings highlight the Cerrado’s critical but undervalued role in global biodiversity, its vulnerabilities, and the urgent need for conservation to avoid irreversible species and biome loss.

## Abstract (web summary)

The South American Cerrado has an estimated ~1605 tree species, yet > 2% make up half of all trees. Since 1995, the savanna lost 24 billion trees, threatening its biodiversity. Urgent conservation is needed to prevent irreversible loss.

## Introduction

South America harbors, by far, the Earth’s largest tree flora^1^, especially due to the contribution of the Amazon and the neighboring biomes. South America’s Cerrado savannas cover two million km^2^, and border four other megadiverse realms including Amazonia. As the world’s most biodiverse, endangered, and deforested savanna, the Cerrado is a critical biodiversity hotspot^2,3^, and as the Brazilian “Cradle of Waters”^4–6^, the Cerrado can simultaneously support urban water demands, while serving as a protective barrier against the agricultural frontier and climate change encroaching upon Amazonia^7^. The two biomes are now largely separated by an anthropogenic deforestation barrier, creating distinct ecotones and abrupt ecosystem change^4–7^. This Cerrado-Amazon transition zone is also an area of intense agrarian conflict, with the advance of the agricultural frontier encroaching on public and indigenous lands.

The whole Cerrado remains chronically neglected and vulnerable, with a lack of international attention, having already lost nearly half its vegetation^8^ while conservation units only safeguard 8% of its extent^9^. Despite estimates of ~12,000 vascular plant species, of which ~4400 are endemic and ~1800 are trees^10,11^, we have very poor knowledge of Cerrado tree density, composition and abundance across both space and time^12,13^. This not only hinders formulation of effective conservation and management strategies but undermines our ability to understand biodiversity and ecological processes in the Cerrado and the Amazon-Cerrado transition. Conservation efforts for the Cerrado should match those in the Amazon since climate change and intensified human impacts could lead to tree species loss and trigger extinction events^8,14,15^, both here and in the adjacent southern edge of the Amazonia^7^.

For complex tropical biomes, high-volume, high-quality well-distributed community sampling is essential to understand species-abundance patterns and ecosystem geography. This approach has already shown that less than 2% of the Amazon’s estimated 16,000 species comprise half of all individual trees^16,17^. Here, by developing and analyzing the most intensive biome-wide quantitative tree sampling to date, we provide a detailed assessment of whether such extreme ‘hyperdominance’ also exists in its giant savanna neighbor to the south. Our study also addresses additional fundamental but unanswered questions about Cerrado trees: How many trees are there? What is the total number of tree species? Which are the most common, and to what extent do they dominate? Finally, we aim to understand how the individual distributions of these species define the geographic limits of the Cerrado. Each of these knowledge gaps hinder progress on key ecological and policy concerns, including resolving contrasting expectations regarding projected Cerrado and Amazonia expansion and retraction (*viz*., savannization^18^ or forest expansion^5,19^), assessing the effect of anthropogenic actions on species composition^20^, projecting fire and extreme drought impacts on the resilience, resistance, and persistence of the Cerrado^21^, and, of course, informing Cerrado conservation efforts (e.g., refs. 7, 15, 22).

**Fig. 1:**
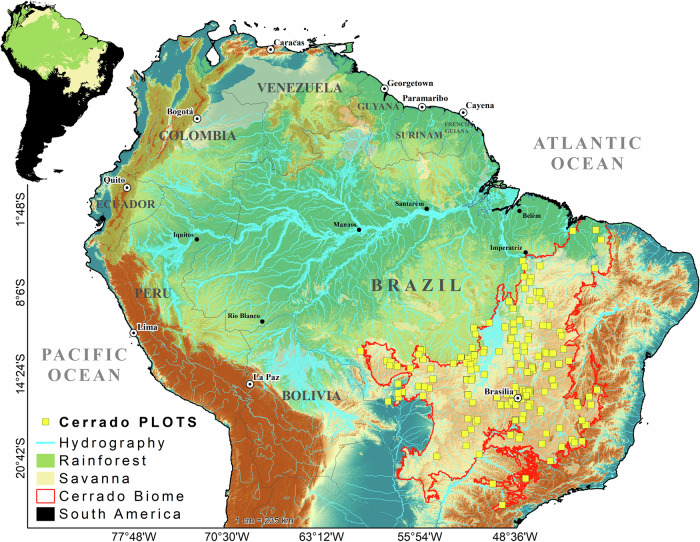
Location of the 222 plots that contributed data to this manuscript. Geographic distribution of savanna (light ochre) and forest (green) formations in South America, combined with the maximum extent of annual flooding (light blue) and digital elevation model (color gradient from blue to brown). The geographic distribution of Cerrado (red line) plots are represented by yellow squares.

To address these critical gaps, we assembled a unique, ground-sourced, biome-wide ecofloristic dataset, and explored it to provide the first, spatially-explicit assessment of commonness and rarity of the Cerrado tree flora. We characterize the dominance, rarity, population sizes, and geographic distributions of tree species representing the Cerrado savanna, referred to as the ‘true Cerrado vegetation^20^, by assembling a unique 222 inventory plot dataset that syntheses decades of research across the biome (Fig. 1) and applying inverse distance weighting to spatiotemporally interpolate the abundances of tree species. Subsequently, we analyze regional dominance by dividing the Cerrado into biogeographic districts, creating a ranked abundance distribution for each district, and comparing the vegetation cover pixel-by-pixel of 1985 and 2020 to estimate population size variations. Through this analysis sequence, we estimate the total number of trees (DSL30 ≥ 5 cm), the average density of trees, the total and relative abundance of species, and the spatiotemporal variations in tree dominance of the Cerrado savanna.

## Methods

### Study area

The Cerrado and Amazonia (Fig. 1) are the largest biomes in South America, representing, respectively, the largest and most tree-biodiverse savanna and tropical rainforest in the world^9,10^. They are currently largely separated by an anthropogenic deforestation barrier, creating distinct ecotones and transitions with other adjacent biomes^4–7^. The transition between the two is an area of intense agrarian conflict, involving the advance of the agricultural frontier and the encroachment on public and indigenous lands^22,23^.

The Cerrado Biome, first defined by Martius in 1943, presents a shrub-arboreal vegetation with phytophysiognomies that include forest, savanna, and grassland formations^24,25^. We analyzed data collected exclusively in the savanna phytophysiognomies of the Cerrado *stricto sensu* and its subdivisions (Cerrado denso, Cerrado típico, Cerrado ralo, and Cerrado rupestre)^24,25^. The Cerrado covers 22% of Brazil’s surface, ~1.5 million km² represents the core area, and this value increases to ~2 million km² when considering the peripheral zone^25–27^. The latitudinal extent of the Cerrado and its borders with other biomes impart a seasonal climatic dynamic, with annual rainfall ranging from 600 to 800 mm along the Caatinga border, and exceeding 2000 mm at the Amazonia border^28^. The climate of the Cerrado, according to the Köppen classification, is predominantly tropical wet (Aw), with variations of humid subtropical climate (Cwa) in higher altitude regions and average annual temperature values oscillating between 18 °C and 28 °C^28^. Generally, the soils of the Cerrado savanna are well-drained and weathered, originating from the Tertiary Period, predominantly acidic, with high concentrations of Al, low nutrient availability, and composed of kaolinite, goethite, and gibbsite, varying regionally between Oxisols, Podzolics, and Argisols^29,30^.

### Data sources

We utilized vegetation inventory plots located within the geographic boundaries of the Cerrado (MAPBIOMAS v.7.0: [https://mapbiomas.org]), including some located less than 50 km from the Cerrado’s outer limits (Fig. 1). Our study focused on self-supporting woody-stemmed plants (hereafter referred to as ‘trees’) with a minimum diameter of 5 cm at 30 cm above the ground (DSL_30_)^31^, situated in areas not affected by floods or water table fluctuations. We incorporated 1 ha plots compiled from the literature and various laboratories and research centers, representing an unprecedented regional collaborative effort in the Cerrado (Supplementary Table 1). Out of a total of 764 studies reviewed, only 120 met our specific criteria, yielding a final tally of 222 plots, including those from virtual platforms (Supplementary Table 1). Once the general database of 222 plots was consolidated, listing all species with corresponding abundance values, we undertook a rigorous editing process: 1) using the Reflora platform (https://reflora.jbrj.gov.br/reflora), we corrected species names for identification and synonymy errors, also verified by the “Taxonomic Name Resolution Service” (TNRS v3.2: http://tnrs.iplantcollaborative.org); 2) we disregarded varieties and subspecies, identifying all individuals at the species level; and 3) we excluded domestic/exotic species and those classified as subshrubs or lianas.

### Statistics and reproducibility

To estimate tree populations in the Cerrado, we employed the methodology developed by ter Steege et al.^16,32^ for Amazonia. This method involves the use of loess regression or inverse distance weighting to spatially interpolate species’ relative abundances. These abundances, combined with a similar interpolation of the total tree count, provide an estimate for the total number of individuals per species. The challenges posed by the low number of plots and their non-random geographic distribution were addressed by conducting 1000 repetitions of a bootstrapping exercise^16,17^. This approach has proven effective in generating stable and reliable estimates of tree populations, regardless of sampling effort and geographic bias due to spatial autocorrelation. To estimate average tree density, we divided the Cerrado’s 2 million km² into 0.5° cells (DGCs) and applied inverse distance weighting (IDW), using only geographic occurrence data, with latitude and longitude as independent variables and a span of 0.5 (see refs.^16^, ^33^). We then estimated the total number of trees (DSL30 ≥ 5 cm) in the Cerrado using IDW on tree density (individuals per hectare) from the 222 plots, as above. We adjusted the total number of trees by correcting for the size of grid cells based on the distance from the Equator.

To determine the total abundance of tree species in the Cerrado savanna, we employed a second, similar model for the 585 identified species, augmented with a bootstrapping exercise to align the mean abundance intervals with the Fisher log series distribution (Supplementary Fig. 1). Thus, we converted the species abundances in each plot to relative abundances: RAi = ni/N, where ni is the number of individuals of species i, and N is the total tree count. For each of the 585 species, we developed a local RAi regression model, incorporating the interaction between latitude and longitude as independent variables and a span of 0.5 (see refs. 16, 33). The relative abundances predicted by these local regression models for each DGC allowed us to map the spatial variations of each species across the Cerrado (Supplementary Fig. 2). We calculated the total population size for each species by multiplying its relative abundance in each DGC by the total number of individuals per DGC, then summing these figures across all 222 plots.

**Fig. 2:**
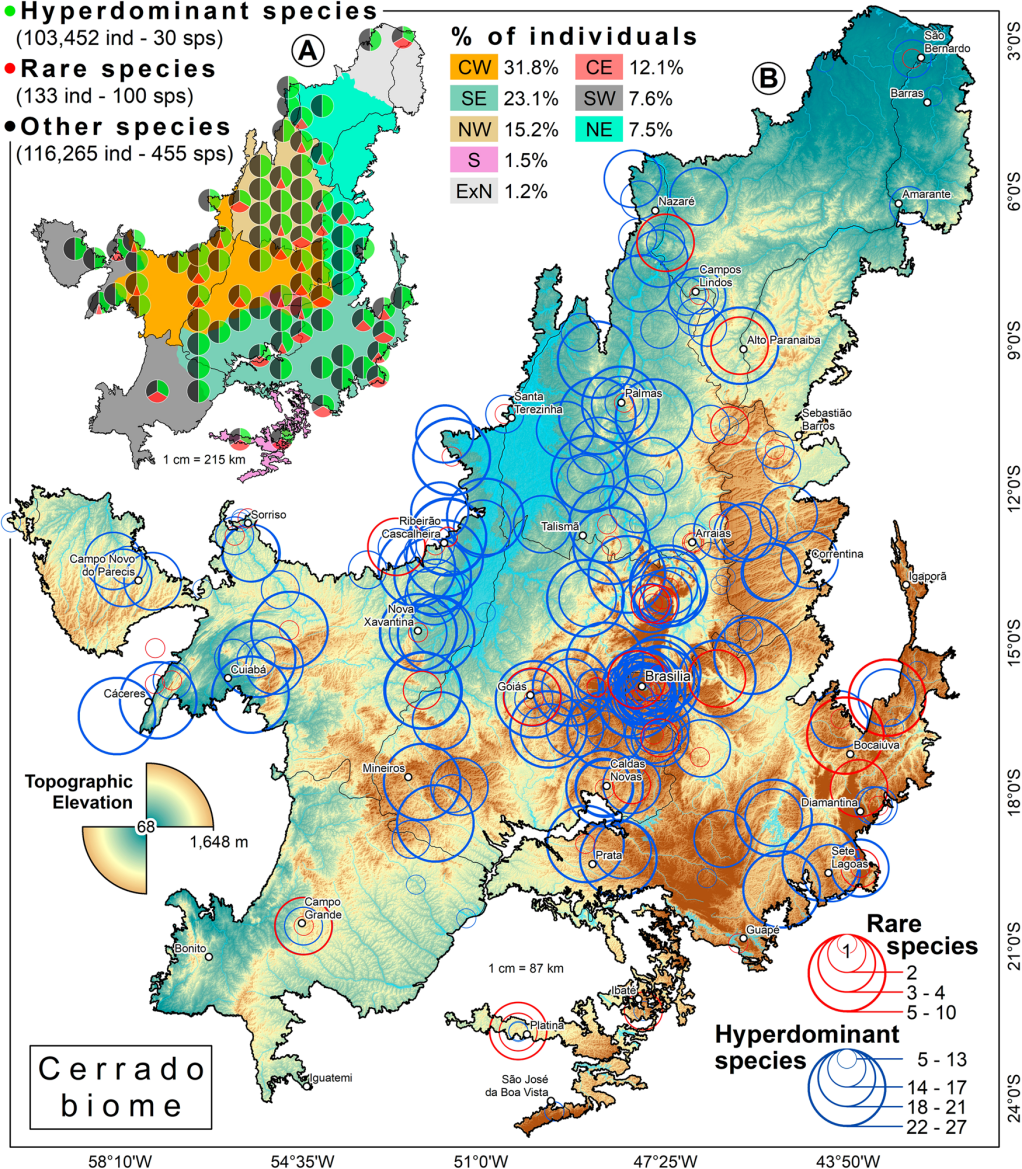
Geographic proportions of Cerrado savanna trees. **A** Geographic distribution of proportions of tree species [(hyperdominant (green), rare (red) and other species (black)] within the geographic areas of the Cerrado and the percentages of individuals registered for each biogeographical domain. **B** Digital elevation model and geographic distribution of relative abundances of hyperdominant (blue circles) and rare (red circles) tree species of the Cerrado savanna. The biogeographical domains represented are: Central-West (CW), Central Brazilian Plateau (CE), Northeast (NE), Northwest (NW), North (ExN), South (S), Southeast (SE), and Southwest (SW), proposed by Françoso et al.^26,40^.

We assessed regional dominance by dividing the Cerrado into eight biogeographical districts (BD) (Fig. 2A), as suggested by Françoso et al.^26^ and created a ranked abundance distribution (RAD) for each BD. We then aggregated the population sizes within each DGC where the species were recorded (Supplementary Fig. 2). Species were considered hyperdominant if they accounted for 50% or more of the RAD. Models utilizing latitude and longitude as predictors produce conservative estimates that minimize overestimation (type I errors)^16^. In this case, we chose a span of 0.5° to reduce the risk of overestimating in areas without known species presence. From the Figshare platform, we share the input data (not raw) and the code to ensure the reproducibility of the analyses and figures (*67*: 10.6084/m9.figshare.28020971.v1).

To analyze the spatio-temporal vegetation dynamics within the Cerrado Biome, we used two raster datasets with the highest temporal resolution for the Cerrado, one from 1985 and another from 2020^34^. Within the actual area of each cell, we extracted vegetation cover pixels for the years 1985 and 2020, calculated the difference between the two periods, and estimated the population size by grid cell (see ref. ^35^ for more details).

### Reporting summary

Further information on research design is available in the Nature Portfolio Reporting Summary linked to this article.

## Results

### Identification and dimensions of Cerrado hyperdominance

Four key results emerge from our synthesis and analysis. First, of the original ~2 million km^2^ Cerrado, 70% was covered by savanna ( ~ 1.4 million km^2^) and we estimate that it originally hosted 141 billion trees, at a density of 1007 trees per hectare greater than 5 cm diameter^36^. Second, by examining the rank abundance distribution (RAD) of modelled populations, we found that just 30 (1.9%) of the estimated 1605 species accounted for half of all trees in the Cerrado savanna (RAD > 50%); these select few are thus the Cerrado hyperdominants^16^ (Table 1, Fig. 2B).

**Table 1:** The 30 hyperdominant trees in the world’s most diverse savanna

Hyperdominant species	Mean estimated population (1985)	Estimated population (2020)	N individuals in plots	Plot occurrence (%)
*Qualea parviflora*	5.23E + 09	4.07E + 09	15,752	89.2
*Qualea grandiflora*	3.34E + 09	2.74E + 09	8759	90.1
*Curatella americana*	2.48E + 09	1.92E + 09	5197	53.1
*Pouteria ramiflora*	1.72E + 09	1.41E + 09	4447	76.6
*Tachigali vulgaris*	1.51E + 09	1.23E + 09	3787	59.9
*Davilla elliptica*	1.32E + 09	9.39E + 08	5728	60.8
*Hirtella ciliata*	1.15E + 09	9.89E + 08	1242	13.1
*Ouratea hexasperma*	1.11E + 09	8.78E + 08	6076	60.4
*Byrsonima coccolobifolia*	1.03E + 09	8.06E + 08	3572	81.5
*Byrsonima pachyphylla*	1.01E + 09	7.75E + 08	3654	54.0
*Kielmeyera coriacea*	9.25E + 08	7.19E + 08	4218	69.4
*Salvertia convallariodora*	8.72E + 08	6.92E + 08	1862	59.5
*Vatairea macrocarpa*	8.49E + 08	6.78E + 08	2207	63.5
*Lafoensia pacari*	8.26E + 08	6.45E + 08	2560	74.3
*Hymenaea stigonocarpa*	7.86E + 08	6.30E + 08	2486	76.6
*Byrsonima crassifolia*	7.19E + 08	6.15E + 08	1263	32.0
*Plathymenia reticulata*	7.19E + 08	6.30E + 08	1599	56.8
*Eugenia dysenterica*	7.16E + 08	5.84E + 08	1948	44.6
*Connarus suberosus*	7.15E + 08	5.66E + 08	2334	74.3
*Bowdichia virgilioides*	6.42E + 08	4.93E + 08	1835	75.2
*Caryocar brasiliense*	6.26E + 08	4.78E + 08	2790	63.5
*Roupala montana*	6.00E + 08	4.31E + 08	3131	71.6
*Xylopia aromatica*	5.73E + 08	4.12E + 08	2103	51.8
*Qualea multiflora*	5.56E + 08	4.09E + 08	2278	60.8
*Aspidosperma tomentosum*	5.16E + 08	3.96E + 08	2689	61.7
*Terminalia argentea*	5.01E + 08	3.47E + 08	1182	34.2
*Astronium fraxinifolium*	4.67E + 08	3.52E + 08	1297	41.0
*Dalbergia miscolobium*	4.49E + 08	3.52E + 08	2944	48.2
*Erythroxylum suberosum*	4.42E + 08	3.33E + 08	2597	54.9
*Stryphnodendron adstringens*	2.89E + 08	2.29E + 08	1915	51.8

Estimated average population sizes, with corresponding empirical data on their Cerrado abundance and frequency.

**Fig. 3:**
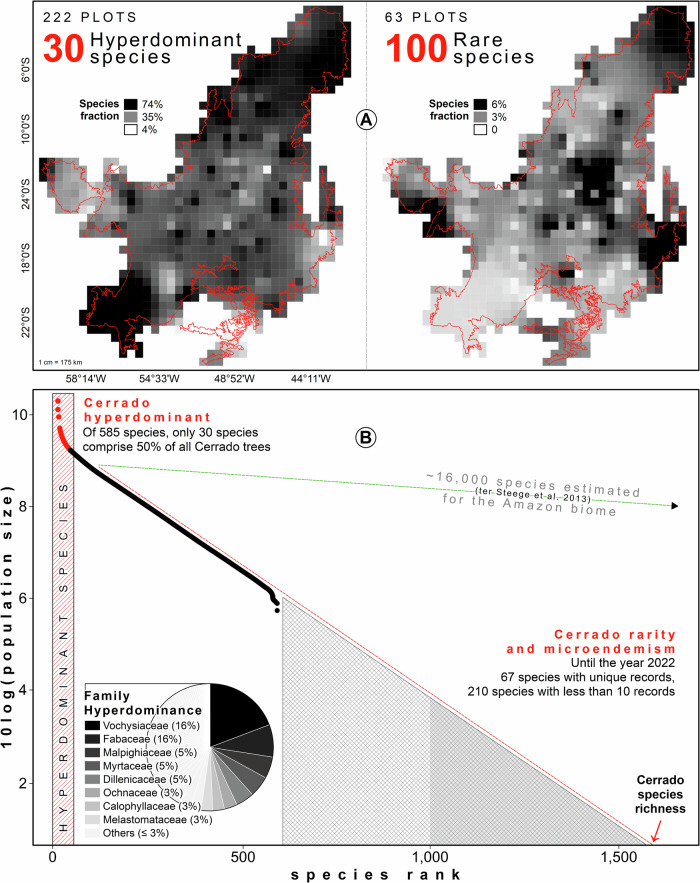
Densities, rank abundance distribution of hyperdominant and rare species. Distribution of the degree of dominance of hyperdominant species (recorded in 222 plots) and rare species (recorded in 63 plots) across the Cerrado (**A**). Complemented by the rank abundance distribution for the 585 tree species recorded in the Cerrado savanna (**B**). The dashed lines represent the species richness estimates for the Cerrado (red line) compared to the Amazonian (green line; ter Steege et al. 2013).

There are strong similarities between theoretical models of Cerrado tree species richness^9,26,37–39^ and our species abundance distributions based on empirical data. Third, alongside extreme dominance by a few, the Cerrado savanna includes a large number of rare species with heterogeneous distributions (Fig. 2). Of these, 371 species (63.4% of the total) have less than 100 trees across the 222 plots, 100 species (17.1%) were represented by only one or two records, while 67 were unique in our dataset (11.4% of all species and 0.03% of all trees) (Fig. 2, Fig. 3). For the first time, we estimated 800 species potentially unknown to science, which could vanish within a few decades, given the current rates of deforestation.

Fourth, our analysis, using remote sensing, revealed that since 1985, the Cerrado has lost 23.9 million hectares of savanna vegetation, therefore ~24 billion savanna trees, an amount equivalent to three times the Earth’s human population, thereby posing severe risks to both ecosystem services and species survival. When evaluating the distribution of individuals in biogeographic districts of the Cerrado^26,40^, we recorded the highest percentage in the Central-West district, with 31.8% of all individuals (Fig. 2A). This heterogeneous loss involves the land conversion of all types of savanna phytophysiognomies of Cerrado into crops and pastures.

Across all plots we measured 219,850 trees, representing 585 species from 76 families (Supplementary Table 1). The most dominant families were Vochysiaceae (37,261 individuals; 21 species), Fabaceae (35,393; 96), Malpighiaceae (12,791; 22), Myrtaceae (11,903; 63), Dilleniaceae (11,036; 3), and Ochnaceae (7280; 6) (Supplementary Table 2), collectively accounting for more than 50% of all trees in the Cerrado savanna. Fabaceae, the second-most abundant family, exhibited the highest species richness (16.4%). In contrast, Cunoniaceae, Lacistemataceae and Thymelaeaceae were each represented by a single individual. *Qualea* was the most dominant genus, and included the top two most common species of the Cerrado savanna (Table 1).

## Discussion

### Probable causes and consequences of hyperdominance

The dominance structure of the Cerrado and Amazon arboreal communities are remarkably similar, with 1.9% of species hyperdominant in Cerrado and 1.4% in Amazonia, despite a 10-fold difference in the number of tree species^16^. On an area basis, however, the Cerrado, an area equivalent to one-third of the Amazon Biome, has proportionally fewer hyperdominant species^16^. This implies that hyperdominants in the Cerrado biome domain occupy a broader geographical range on average compared to their equivalents in the Amazon, despite the similar level of hyperdominance in relative terms between the two biomes and the fact that Amazonia is three times as large. In both, the great majority of tree species are rare. The causes of extreme dominance by a few species in the presence of such diversity are poorly understood. Neutral processes have been suggested, but in both biomes, the dominant trees are more dominant than neutrality predicts^16,41^. For Amazonia, it has been suggested that resistance to pathogens or domestication by pre-Colombian cultures could explain dominance by the few^42–44^. For the Cerrado, tree hyperdominance is more likely associated with niche differentiation under extreme climate (rainfall seasonality, heat waves), soil conditions (dystrophic, acidic and allic), and fire (Supplementary Text 1), rather than with biological factors such as resistance to pathogens, herbivory, and other sources of density-dependent mortality^26,37,45–48^. In contrast to forests, savannas lack a continuous canopy and understory, leading to less competition for light among tree species and therefore potentially less differentiation in terms of light preference. Moisture, temperature and nutrient restrictions are more likely to act as robust filters for tree species, potentially exacerbated by human presence. Widespread pre-1492 fire use by South American indigenous people was likely even more prevalent for the Cerrado^49^ than for Amazonia^42,50^, and implies a partial anthropogenic explanation for hyperdominance by enhancing the species filtering effect beyond that due to infrequent natural lightning-induced fires^51^.

Of an estimated 1605 tree species in the Cerrado savanna, just 30 comprise half of all trees, with *Qualea parviflora* being the most common and widely distributed, and especially dominant in the southern Cerrado. Vochysiaceae was the most dominant family in terms of the number of individuals. Low soil fertility, high toxic aluminum (Al) concentration, and fire are the primary limiting factors frequently associated with the distribution of tree flora in the Cerrado^52^. The prevalence of dystrophic soils with a high concentration of toxic Al (allic) in the Cerrado selects particular groups of Al accumulators^52^. Remarkably, 80% of the species identified in this study as hyperdominant are also Al-hyperaccumulators, with *Qualea parviflora* exhibiting the greatest dominance and accumulation of Al within the Vochysiaceae^52^, a pattern that is unlikely to be merely stochastic. Some species are not only tolerant to Al but also dependent on it; accumulating aluminum in leaves ( >1000 ppm) can offer competitive advantages due to the potential increase in resistance to pathogens and herbivory provided by Al toxicity^53^. Furthermore, in the specific case of *Q. grandiflora*, Al enters the chloroplasts without causing significant damage to these organelles^53^. Fabaceae, the next most dominant and diverse family, similarly dominates lowland South American forests^54^. Family dominance varies with biotic and abiotic interactions, such as herbivory, pathogens, soil, climate, and water availability, factors that potentially drive their abundances^33,45,46^. For example, Dilleniaceae, particularly represented by the Cerrado hyperdominant *Curatella americana*, accumulates silicon from the soil in its leaves, enhancing resistance to drought, fungi, and insects^35^, a factor that can contribute to its dominance.

We estimated the original tree population of the Cerrado savanna at ~141 billion. We recognize that the abundance of a common species is proportional to the probability of correct identification. Hence, the relative abundances of the 30 species identified here as hyperdominant suggest that few (or none) of these have taxonomic errors to be misclassified as hyperdominant. Notably, 67 species recorded in this study were found only once, representing 11% of all species and 0.03% of all trees. This underscores that the full range of species richness and abundance in the Cerrado remains incompletely explored, with only 36.4% (585 species) of the estimated 1605 species discovered in this study. Such a condition reveals the vulnerability of these species to extinction, a situation similar to that recorded for the Amazon^16^. These findings highlight the importance of conserving both hyperdominant and rare species to safeguard the ecological integrity, ecosystem functioning and diversity of the Cerrado savanna.

Hyperdominance appears to depend on location (Supplementary Text 2). This phenomenon is particularly evident in certain extreme cases of monodominance^33,46^. Geographical factors may also play a role in hyperdominance, where the number and ranking of hyperdominant species could change according to spatial scale and biogeographic districts^40^ (Fig. 2). This study represents the first comprehensive analysis of tree species dominance and abundance across the Cerrado. The macroecological scope of this study, coupled with standardized sampling from extensive fixed-area ecofloristic inventories, offers regional scale insights and deeper understanding, allowing us to estimate that the world’s most diverse savanna is composed of 1605 tree species. It also reveals a significant gap in our knowledge of the Cerrado, highlighting the necessity to systematize scientific efforts within a collaborative network to enhance global savanna databases.

### Practical implications of hyperdominance and rarity

The practical implications of hyperdominance and rarity in the Cerrado Biome extend beyond its scientific significance to encompass ecological and conservation concerns. The Cerrado currently faces two major threats. The first is related to climate change; our analysis suggests enhanced risk due to functional concentration within a few hyperdominants, which dominate the ecosystem structure. Should global warming and increasing seasonality continue at the current rate, and if hyperdominant species are unable to adapt to the rapidity and intensity of these changes, ecosystem functions could be severely compromised. The unequal geographical distribution of hyperdominant species could also help identify and prioritize regions for biodiversity conservation to reduce extinction risks, such as in the Central-West areas of the biome (for further results on hyperdominant species, see Figs. 2, 3). This region, with the highest abundance of individuals and hyperdominant species, is not only experiencing the most direct human intervention but also may face the greatest climate-driven threat to the loss of ecosystem function.

The second major threat is the risk of species extinction due to deforestation. The Cerrado lost approximately 24 million hectares between 1985 and 2020 (35 years). Considering the biome’s original conservation condition decades before 1985, the Cerrado has experienced a loss of approximately 50% of its total area of native vegetation. If deforestation rates like those in 2023 (782,800 hectares)^55^ persist, this could result in the loss of ~80% of Cerrado savanna vegetation within the next 50 years. Such a scenario could trigger one of the planet’s largest species extinctions, given this biome’s high endemism and the extremely high number of rare species (for further results on rare species, see Figs. 2, 3). Furthermore, we estimate that the majority of the approximately 800 unknown tree species implied in this study might vanish before being scientifically documented. Between 1985 and 2020, the Cerrado lost 24 billion trees, equivalent to three times the Earth’s human population, posing severe threats to ecosystem services and species survival. This loss is exacerbated by Brazilian legislation (Forest Code), which permits deforestation of up to 80% of the biome’s savanna vegetation for agriculture and livestock, vital for the country’s economy.

The Cerrado plays a crucial role as the gateway to Amazonia and other biomes, so its deforestation also threatens the integrity of bordering biomes. To date, it has acted as a barrier, helping to separate Amazonia from the densely populated and urbanized southeastern region of Brazil, but the losses have been immense. As approximately 50% of the Cerrado has been converted, following the species-area curve this implies an eventual species loss of ~10%. The resilience of its trees is currently being surpassed by anthropogenic actions^8^. The mechanisms governing species dominance, rarity, and distribution, and hence their risk of extinction, remain largely unknown here^33^ (for further results, see Supplementary Fig. 2). As only 30 species dominate the Cerrado, its stability and functioning are very narrowly based. To help safeguard the biome as well as halt land conversion we urgently need to improve our understanding of these species. While our results provide biome-wide estimates of tree species richness and abundance in the Cerrado savanna, contributing to addressing the Prestonian deficit^12^, significant knowledge gaps persist including two extensive zones lacking data. These too must be priorities for future work (Fig. 3 and Fig. 2A).

The preservation of large-scale ecosystem services offered by Amazonia and the Cerrado, such as CO_2_ sequestration and global thermoregulation (valued between US$ 1.5–3 trillion/year), freshwater supply ($2 quadrillion/year), and biomass renewal by biotic decomposition (US$ 5–13 trillion/year), demands global action^22^. Future research focusing on palynological records, paleoclimatic responses, and molecular phylogenetics could provide new insights into the biogeographic origins and future trends of the Cerrado savannas. While global efforts, such as international conservation agreements and research collaborations, are focused on protecting the Amazon, the Cerrado remains a neglected biome. Its conservation should be a focus for global interest and participation and not a burden for local and native communities. Tackling the funding, scientific, and public awareness gaps the Cerrado faces is crucial not only to understand it but also for effective conservation of the world’s largest and most biodiverse savanna ecosystem. International and cross-sectoral collaboration will be essential to preserve the Cerrado and mitigate climate change through targeted conservation strategies and sustainable management practices.

## Supplementary information

Supplementary Information

Reporting Summary

## Data Availability

All data are available in the main text (Supplementary Table 1) or the supplementary information (10.6084/m9.figshare.28020971.v1).

## Code availability

Codes and data (not raw) used to ensure reproducibility are available in the Figshare public repository (10.6084/m9.figshare.28020971.v1).
