# Supplementary material for: Tree species hyperdominance and rarity in the South American Cerrado
Source: Commun Biol. 2025 May 3;8:695. doi: 10.1038/s42003-025-07623-w (PMC12049495; doi:10.1038/s42003-025-07623-w)
Supplement: Supplementary file 1 — Supplementary Information [file 42003_2025_7623_MOESM1_ESM.pdf]

Supplementary Information

Supplementary Figures

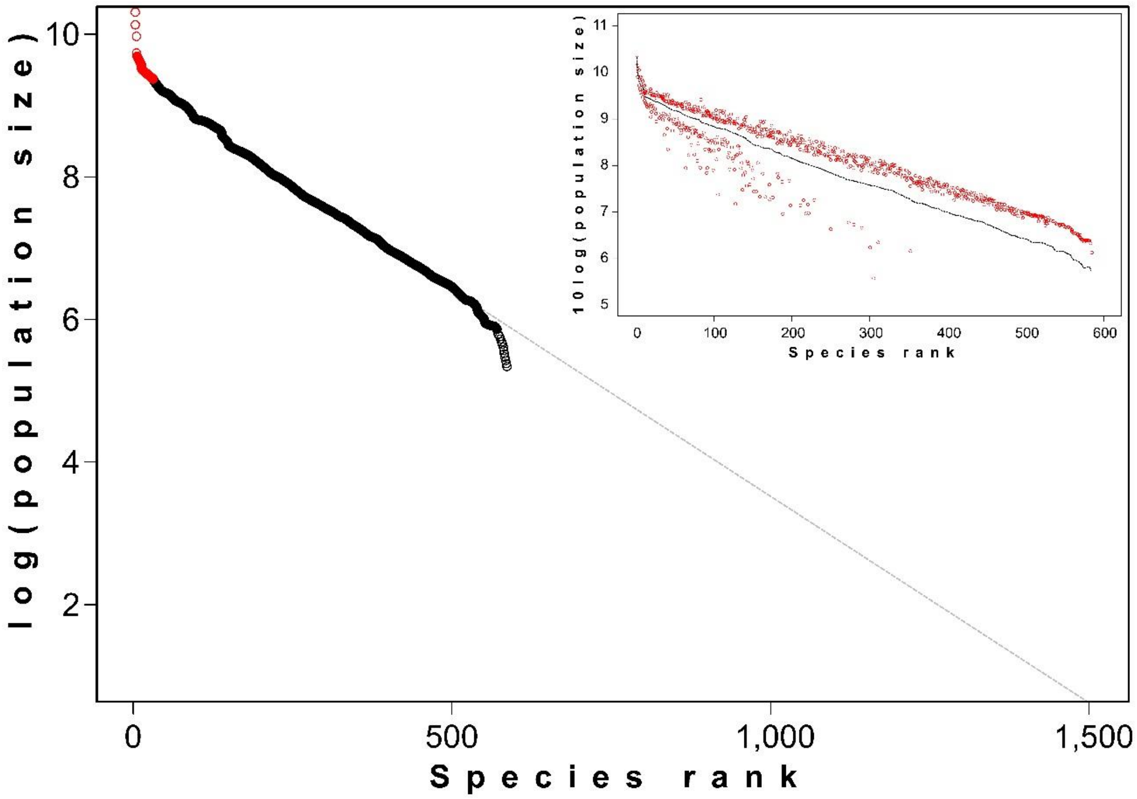

Supplementary Figure 1. Estimated ranked abundance distribution (RAD) for the Cerrado savanna. RAD values obtained from 500 starters exercises and 95% confidence intervals for 585 known species (DAS30 ≥ 5 cm) and 1,605 estimated.

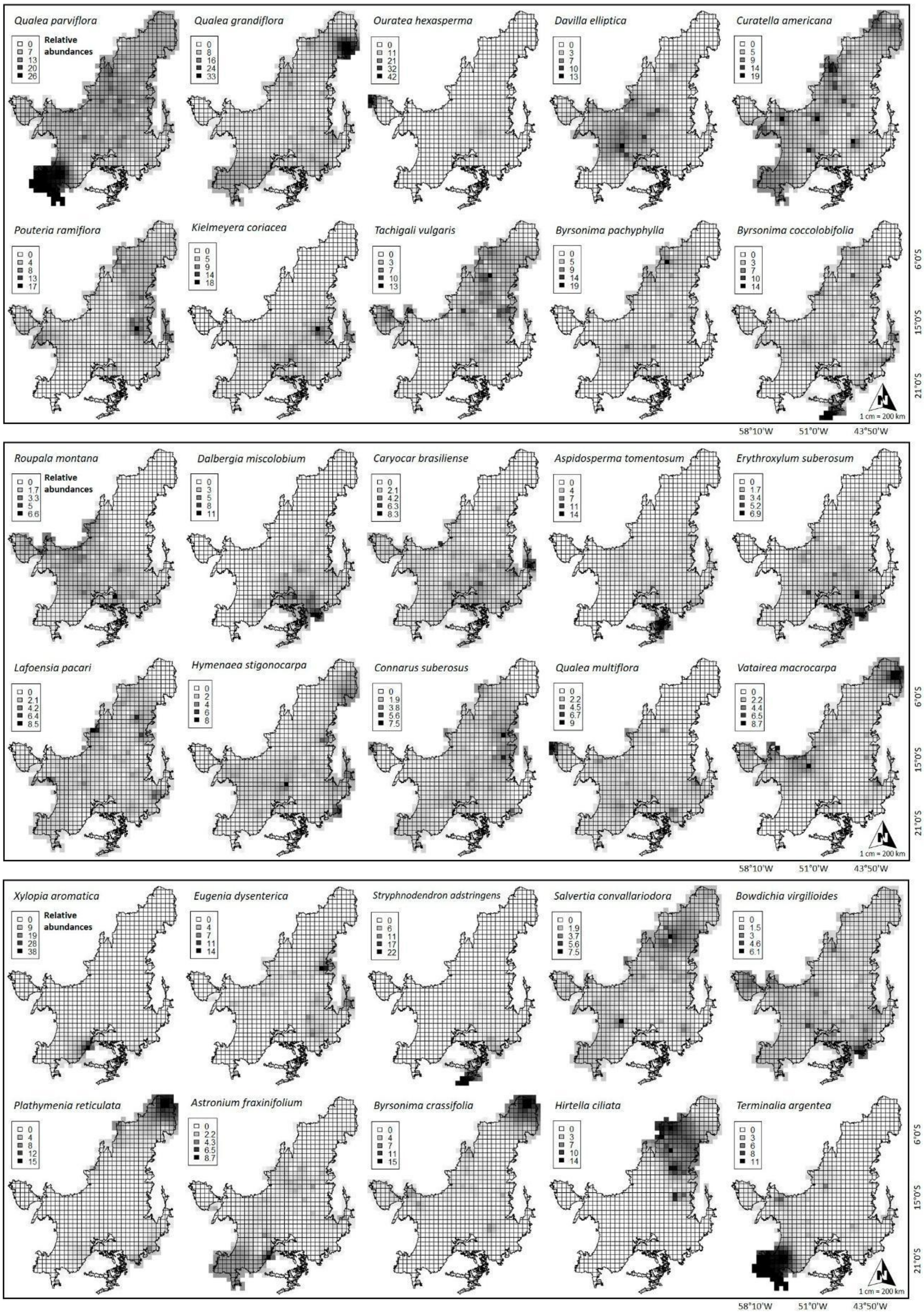

Supplementary Figure 2 (A - C). Geographic abundance distribution. In gradient from white (lower values) to black (higher values) spatial pattern of responses for the relative abundance of each hyperdominant species in the Cerrado savanna.

Supplementary Text

Supplementary Text 1: Fire, traditional communities and hyperdominance of the Cerrado

If humans partially defined Amazonian hyperdominance, did fire do this in the Cerrado? While the hyperdominance of the Amazonia partially responds to historical anthropogenic interventions (traditional communities' architects of the forests), the hyperdominance of the Cerrado may be subject to long-term drought and fire historical events (*37*, *29*). Although these events are natural and stochastic, fire has been used in South America for over 10,000 years (*36*, *59*), being one of the environmental and anthropic architects of the Cerrado (*60*).

The hyperdominance within the Cerrado Biome exhibits a core-periphery pattern, with hyperdominance cores closely associated with the Cerrado's topographic features, notably the Chapada do Rio São Francisco (Central-East: CE and Southeast: SE), Serra do Roncador (Central-West: CW), Central Brazilian Plateau (CE), Alto Rio Parnaíba Plateau (North: N and Northeast: NE), Mountains and Plateaus of the Tocantins and Araguaia rivers (Northwest: NW), Chapada and Planalto dos Parecis (Southwest: SW), and the Plateau of the Paraná River in the southern region (Fig. 2). Besides the core-periphery pattern, we observed geographic patterns of selective species or habitat specialists. For instance, extreme and exceptional dominance in the southern part of the Cerrado Biome was recorded for *Qualea parviflora*, *Byrsonima coccolobifolia*, and *Terminalia argentea* (Extended Data Figure 2). Other species, like *Qualea grandiflora*, *Vatairea macrocarpa*, *Plathymenia reticulata*, and *Byrsonima crassifolia*, showed selectivity for the northern region (Extended Data Figure 2). Generalist species, with widespread and hyperdominant habitats across the Cerrado, included *Curatella americana*, *Salvertia convallariodora*, and *Bowdichia virgilioides* (Extended Data Figure 2).

Of the top 20 hyperdominant species listed by ter Steege et al. (*16*) for the Amazon, 85% have fruits, seeds, or stems used to feed traditional communities, reinforcing the role of humans in the hyperdominance of Amazonian species (*29*). In the case of the 20 main hyperdominant species in the Cerrado, 25% are directly used in the diets of traditional communities within the Cerrado savanna (*61*), reinforcing the idea that these may be environmental elements shaping the pattern of species hyperdominance in the Cerrado (Table 1). The 23% of the hyperdominant species identified by us for the Cerrado Biome have fruits consumed by traditional communities of the Cerrado (*62*), such as the Xavante and the Xerente (*63-65*). With emphasis on the Pequi (*Caryocar Brasiliense*, 21st) whose populations decreased from 6.26E+08 in 1985 to 4.76E+08 in 2020. If this trend continues, different traditional communities will lose their main food resources. The other hyperdominant species may represent resources that we currently ignore. For example, *Byrsonima verbascifolia* and *B. crassifolia*, both hyperdominant, have been mentioned by different traditional communities as medicinal species (*63*). In the leaves, trunk, and bark of the two main hyperdominant species of our study, *Qualea parviflora* and *Q. grandiflora*, the presence of flavonoids, triterpenoids, steroids, tannins, benzoquinones, and anthraquinones has already been recorded, and antimicrobial activity was verified (*66*).

Supplementary Text 2: Geographic patterns of hyperdominance and rarity

In addition to the core-periphery pattern, we report geographic patterns of selective species or habitat specialists. For example, we recorded an extreme and exceptional dominance in the south of the Cerrado Biome for *Qualea parviflora*, *Byrsonima coccolobifolia* and *Terminalia argentea* (Extended Data Figure 2). Other species showed

selectivity for the northern region, such as *Qualea grandiflora*, *Vatairea macrocarpa*, *Plathymenia reticulata*, and *Byrsonima crassifolia* (Extended Data Figure 2). Generalist species, with widely distributed and hyperdominant habitats throughout the Cerrado were *Curatella americana*, *Salvertia convallariodora* and *Bowdichia virgilioides* (Extended Data Figure 2).

Similar to Amazonia (*16*), hyperdominant Cerrado species occupy larger distributional ranges than other taxa (Fig. 2A). The greatest numbers of individuals and species were recorded in the biogeographical districts (BD) proposed by Françoso et al. (*22*) of the Centre-west (CW 30,501; 14%), Southeast (SE 25,988; 12%) and Northwest (NW 12,011; 5.5%) and the highest number of rare species was recorded in the Centre-west (36 sp.) and Southeast (17 sp.) (Fig. 2A). In the ~2 million km^2 originally occupied by the Cerrado only 30 tree species dominated and represented 580,000 km^2 of the Cerrado savanna (29%). In addition to representing a low number, these species present habitat selectivity and are dominant: with 100% dominance in at least two of the BD proposed by Françoso et al. (*22*). Thus, the regionalization of hyperdominance of Cerrado savanna tree species is centered in the CW, SE, and NW districts, with *Qualea parviflora* dominating in six of the eight BDs (13,291 ind.), while *Ouratea hexasperma* (3,081 ind.) was dominant in the SE region and *Stryphnodendron adstringens* (390 ind.) in the southern region.

The geographic distribution of the hyperdominance cores identified here coincides with historical flora refuges and centers of endemism and diversity (*11, 56, 57, 58*). Although the entire Cerrado is considered a biodiversity hotspot (*10*), these hyperdominance cores may represent the actual geographic extent and current geographic positioning of the hotspots that define the Cerrado. In the entire sampled area, the proportion of individuals of hyperdominant species was greater than that of individuals of rare species, but the number of rare species surpassed that of hyperdominant ones. In addition, some Cerrado biogeographical districts had higher proportions of rare species than of hyperdominant species, for example, in the municipalities of Bocaiúva and Diamantina, in Minas Gerais, Platina, and Ibaté, in São Paulo, and Campo Grande, in Mato Grosso do Sul (Fig. 2).

Supplementary Tables

Supplementary Table 1. Plots to characterize the biodiversity of the Cerrado savanna. Abbreviation of the 222 plots, geographic information, and data sources used in the analysis.

Plots	Latitude	Longitude	Sorce
AGB-01	-13.83	-52.00	Present study (Cássia B. R. Munhoz)
AGE-01	-15.52	-47.53	Present study (Cássia B. R. Munhoz)
ALTO	-14.05	-47.51	Present study (Sabrina Miranda)
APG-01	-15.87	-47.83	Present study (Cássia B. R. Munhoz)
ARA-01	-12.94	-46.90	(*67*) (Lemos 2017)
ARA-02	-12.94	-46.94	(*67*) (Lemos 2017)
ARG-01	-5.75	-48.11	Present study (Ricardo F. Haidar)
ATP-01	-14.00	-47.33	Present study (Cássia B. R. Munhoz)
BAL-01	-8.48	-46.87	Present study (Fabiana de Goes Aquino)
BAL-02	-8.68	-46.63	Present study (Fabiana de Goes Aquino)
BAL-03	-10.47	-47.70	Present study (Ricardo F. Haidar)
BAN-01	-8.78	-49.15	Present study (Ricardo F. Haidar)
BAR-01	-12.43	-51.03	(*68*) (Marimon et al. 2008)
CCIP	-15.02	-57.55	(*69*) (ForestPlots.net)
CCR-02	-16.05	-57.49	Present study (Maria A. Carniello)
CE-16	-6.10	-46.81	Present study (Renata F. Brandão)
CE-17	-3.45	-44.33	Present study (Renata F. Brandão)
CE-18	-14.67	-54.90	Present study (Renata F. Brandão)
CE-74	-18.89	-49.05	Present study (Renata F. Brandão)
CE-75	-19.79	-45.17	Present study (Renata F. Brandão)
CE-76	-19.57	-46.65	Present study (Renata F. Brandão)
CHV-01	-14.12	-47.73	(*69*) (ForestPlots.net)
CNR-01	-13.25	-51.83	Present study (Cássia B. R. Munhoz)
COD-01	-6.91	-48.01	Present study (Ricardo F. Haidar)
COR	-13.53	-45.40	Present study (Sabrina Miranda)
CPL-01	-8.07	-47.04	(*67*) (Lemos 2017)
CPL-02	-8.05	-46.80	(*67*) (Lemos 2017)
CR1	-14.16	-47.61	(*69*) (ForestPlots.net)
CR2	-17.80	-48.66	(*69*) (ForestPlots.net)
CR3	-13.79	-47.39	(*70*) (ForestPlots.net)
CR4	-16.79	-47.57	(*69*) (ForestPlots.net)
CR5	-15.79	-49.34	(*69*) (ForestPlots.net)
CR6	-14.03	-49.00	(*69*) (ForestPlots.net)
CR7	-17.66	-52.28	(*69*) (ForestPlots.net)
CR8	-16.68	-49.76	(*69*) (ForestPlots.net)
CR10	-15.80	-48.83	(*69*) (ForestPlots.net)
CRR-01	-13.52	-45.37	Present study (Cássia B. R. Munhoz)
CRX-01	-11.03	-48.79	Present study (Ricardo F. Haidar)
CSE4	-15.66	-57.21	(*69*) (ForestPlots.net)

CT1	-14.14	-47.72	(*69*) (ForestPlots.net)
CT2	-17.76	-48.69	(*69*) (ForestPlots.net)
CT3	-13.75	-47.41	(*70*) (ForestPlots.net)
CT4	-16.73	-47.70	(*69*) (ForestPlots.net)
CT6	-14.04	-49.05	(*69*) (ForestPlots.net)
CT7	-18.48	-52.02	(*69*) (ForestPlots.net)
CT8	-16.62	-49.78	(*69*) (ForestPlots.net)
CT10	-15.80	-48.83	(*69*) (ForestPlots.net)
ESA-04	-15.65	-57.21	(*69*) (ForestPlots.net)
ESA-06	-15.66	-57.22	(*69*) (ForestPlots.net)
ESA-08	-15.66	-57.22	(*69*) (ForestPlots.net)
FAG-01	-12.46	-55.72	Present study (Edson de S. Lima)
FAP-01	-14.32	-51.02	Present study (Edson de S. Lima)
FAR-03	-14.60	-51.93	Present study (Edson de S. Lima)
FBL-01	-13.14	-55.67	Present study (Edson de S. Lima)
FBV-01	-13.03	-52.77	Present study (Edson de S. Lima)
FCO-01	-13.30	-58.37	Present study (Edson de S. Lima)
FCT-01	-13.39	-58.08	Present study (Edson de S. Lima)
FFY-01	-15.36	-55.19	Present study (Edson de S. Lima)
FFZ-01	-12.99	-52.01	Present study (Edson de S. Lima)
FGR-01	-13.70	-57.60	Present study (Edson de S. Lima)
FLG-01	-13.02	-52.78	Present study (Edson de S. Lima)
FMM-01	-13.86	-56.61	Present study (Edson de S. Lima)
FNI-01	-16.51	-47.56	Present study (Cássia B. R. Munhoz)
FPC-01	-13.15	-55.65	Present study (Edson de S. Lima)
FRF-01	-13.87	-55.08	Present study (Edson de S. Lima)
FRP-02	-11.24	-51.69	(*69*) (ForestPlots.net)
FRP-03	-11.10	-45.30	Present study (Cássia B. R. Munhoz)
FRX-01	-13.87	-51.66	Present study (Edson de S. Lima)
FSA-01	-12.54	-55.87	Present study (Edson de S. Lima)
FSH-01	-12.47	-55.85	Present study (Edson de S. Lima)
FSJ-01	-13.14	-52.25	(*69*) (ForestPlots.net)
FVD-01	-13.44	-52.38	Present study (Edson de S. Lima)
GAU-03	-13.48	-53.35	(*69*) (ForestPlots.net)
GNS-01	-15.27	-48.67	Present study (Cássia B. R. Munhoz)
GOV-02	-15.99	-50.09	(*69*) (ForestPlots.net)
GRM-01	-11.40	-45.17	(*69*) (ForestPlots.net)
GRM-02	-16.61	-42.95	(*69*) (ForestPlots.net)
GSV-01	-15.17	-45.75	Present study (Cássia B. R. Munhoz)
INTER	-15.89	-47.83	Present study (Sabrina Miranda)
JAV-01	-12.58	-49.90	Present study (Ricardo F. Haidar)
JBBCV	-15.89	-47.83	Present study (Sabrina Miranda)
LJD-01	-9.32	-48.84	Present study (Ricardo F. Haidar)
LST-01	-16.73	-47.70	Present study (Cássia B. R. Munhoz)
MAN-01	-11.43	-47.70	Present study (Ricardo F. Haidar)

MANSO-01	-15.22	-56.00	Present study (Zenésio Finger)
MARA-01	-12.66	-51.09	(*68*) (Marimon et al. 2008)
MS-01	-20.51	-54.61	Present study (Marcelo Bueno)
MEXU	-15.73	-57.55	(*69*) (ForestPlots.net)
MMA-01	-8.08	-46.84	Present study (Ricardo F. Haidar)
MMA-02	-8.72	-47.58	Present study (Ricardo F. Haidar)
MTE-01	-10.63	-46.21	(*67*) (Lemos 2017)
MTE-02	-10.63	-46.20	(*67*) (Lemos 2017)
NAT-01	-10.22	-48.36	(*69*) (ForestPlots.net)
NXV-01	-14.71	-52.35	(*69*) (ForestPlots.net)
NXV-03	-14.71	-52.35	(*69*) (ForestPlots.net)
NXV-05	-14.71	-52.35	(*69*) (ForestPlots.net)
NXV-10	-14.71	-52.35	(*69*) (ForestPlots.net)
NXV-11	-14.75	-52.00	(*69*) (ForestPlots.net)
OK01-07	-17.78	-48.68	(*70*) (Raymundo et al. 2017)
OK01-19	-20.36	-51.40	(*71*) (Aragão et al. 2013)
OK01-27	-18.13	-43.26	(*72*) (Costa et al. 2021)
OK01-28	-13.59	-52.07	(*73*) (Nogueira et al. 2001)
OK01-36	-15.74	-47.91	(*74*) (Rossi et al. 1998)
OK01-38A	-14.74	-45.92	(*75*) (Oliveira 2014)
OK01-38B	-14.68	-45.86	(*75*) (Oliveira 2014)
OK01-41	-13.78	-46.38	(*76*) (Teixeira 2015)
OK01-45	-15.77	-47.95	(*77*) (Costa 2019)
OK01-47	-15.88	-47.83	(*78*) (Sarmento and Silva-Júnior 2006)
OK01-49	-15.95	-47.92	(*79*) Medeiros et al. (2007)
OK02-06	-15.88	-47.85	(*80*) Haidar et al. (2008)
OK02-21	-11.75	-49.05	(*81*) Tavares (2017)
OK02-27Cui	-15.23	-55.56	(*82*) Oestreich Filho (2014)
OK02-30	-15.95	-47.92	(*83*) Azevedo (2014)
OK02-33A	-24.17	-49.66	(*84*) Uhlmann et al. (1998)
OK02-33B	-24.18	-49.67	(*84*) Uhlmann et al. (1998)
OK02-44	-15.96	-47.92	(*85*) Almeida et al. (2014)
OK03-07Nat-R	-11.69	-47.70	(*67*) (Lemos 2013)
OK03-07Pal-R	-10.17	-48.28	(*67*) (Lemos 2013)
OK03-13	-7.01	-47.47	(*86*) Medeiros et al. (2008)
OK03-22	-16.04	-48.05	(*87*) (Brant 2011)
OK03-32	-17.17	-46.79	(*88*) (Ferreira 2010)
OK03-43	-15.96	-47.83	(89) (Vieira 2018)
OK04-03	-17.79	-48.67	(*90*) (Lopes et al. 2011)
OK04-05A	-15.85	-52.27	(*91*) (Ribeiro et al. 2012)
OK04-05B	-15.86	-52.25	(*91*) (Ribeiro et al. 2012)
OK04-07	-15.98	-50.10	(*92*) (Rios 2018)
OK04-11	-20.86	-46.03	(*93*) (Torres et al. 2017)

OK04-16	-12.58	-46.41	(*94*) (Silva 2011)
OK04-17	-6.27	-42.66	(*95*) Lindoso et al. (2009)
OK04-22	-4.12	-42.18	(*96*) (Lindoso 2008)
OK04-35	-17.39	-48.07	(*97*) (Cardoso et al. 2016)
OK04-38	-15.75	-47.56	(*98*) (Fernandes et al. 2013)
OK04-40	-11.59	-49.03	(*99*) (Silva-Neto et al. 2016)
OK05-08	-16.75	-43.92	(*100*) (Pereira et al. 2020)
OK05-30	-15.47	-42.49	(*101*) (Lima et al. 2012)
OK05-32	-20.51	-54.62	(*102*) (Bueno et al. 2013)
OK05-34	-16.36	-58.31	(*103*) (Morais et al. 2019)
OK05-45	-15.80	-48.82	(*104*) (Moura et al. 2010)
OK05-47	-16.42	-44.00	(*105*) (Campos et al. 2020)
OK05-48A	-17.89	-51.67	(*106*) (Silva et al. 2019)
OK05-48B	-17.76	-51.54	(*106*) (Silva et al. 2019)
OK06-11A	-18.37	-45.33	(*107*) (Giácomo et al. 2013)
OK06-11B	-18.36	-45.32	(*107*) (Giácomo et al. 2013)
OK06-22	-19.01	-48.31	(*108*) (Faleiro 2007)
OK06-25	-17.72	-48.13	(*109*) (Rios et al. 2020)
OK06-40	-19.69	-52.40	(*110*) (Pina et al. 2021)
OK06-43	-9.05	-46.00	(*111*) (Sampaio et al. 2018)
OK06-46A	-16.46	-51.90	(*112*) (Abreu et al. 2012)
OK06-46B	-16.45	-51.90	(*112*) (Abreu et al. 2012)
OK06-48	-16.00	-43.09	(*113*) (Pereira et al. 2013)
OK06-49	-12.57	-47.99	(*114*) (Rêgo et al. 2015)
OK07-08	-15.97	-47.92	(*115*) (Oliveira et al. 2019)
OK07-12	-12.06	-48.58	(*116*) (Coelho et al. 2017)
OK07-14	-15.94	-47.86	(*117*) (Andrade et al. 2002)
OK07-22	-19.25	-44.40	(*118*) (Balduíno et al. 2005)
OK07-41	-16.43	-44.03	(*119*) (Lima et al. 2021)
OK07-45A	-22.60	-50.37	(*120*) (Pinheiro and Durigan 2012)
OK07-45B	-22.58	-50.38	(*120*) (Pinheiro and Durigan 2012)
OK08-02	-18.80	-52.49	(*121*) (Schardong et al. 2020)
OK08-05	-15.88	-47.83	(*122*) (Miranda et al. 2013)
OK08-07	-7.32	-47.59	(*123*) (Medeiros and Walter 2012)
OK08-10	-16.63	-48.66	(*124*) (Araújo et al. 2012)
OK08-12	-15.62	-46.44	(*125*) Nettesheim et al. (2010)
OK08-30A	-13.28	-44.62	(*126*) (Miranda et al. 2017)
OK08-30B	-12.38	-45.03	(*126*) (Miranda et al. 2017)
OK08-32	-12.54	-60.40	(*127*) Miranda et al. (2006)
OK08-38A	-15.64	-48.03	(*128*) (Santana and Imaña-Encinas 2010)
OK08-38B	-16.61	-49.26	(*128*) (Santana and Imaña-Encinas 2010)
OK08-40	-16.19	-48.20	(*129*) (Medeiros et al. 2005)
OK08-49	-16.38	-48.94	(*130*) (Carvalho and Marques-Alves 2008)
OK09-12	-13.63	-58.18	(*131*) (Costa et al. 2014)
OK09-22	-15.53	-47.59	(*132*) (Paula et al. 2007)

OK09-27	-19.46	-44.18	(*113*) (Pereira et al. 2013)
OK09-33	-15.80	-49.33	(*112*) (Abreu et al. 2012)
OK09-46	-15.50	-48.10	(*133*) (Paula et al. 2009)
OK10-02	-22.19	-47.90	(*134*) (Durigan et al. 2002)
OK10-04	-15.95	-47.92	(*135*) (Walter and Guarino 2006)
OK10-07	-13.99	-52.04	(*136*) (Felfili et al. 2002)
OK10-22	-19.17	-48.40	(*137*) (Costa and Araújo 2001)
OK10-29	-3.38	-42.59	(*138*) Imaña-Encinas and Paula (2003)
OK10-30	-15.77	-47.85	(*139*) Assunção and Felfili (2004)
OK10-39	-15.63	-47.94	(*140*) Sinimbu et al. (2007)
PAL-01	-12.70	-46.34	Present study (Ricardo F. Haidar)
PCV-01	-13.83	-47.40	Present study (Cássia B. R. Munhoz)
PEA-01	-12.31	-51.92	(*68*) Marimon et al. (2008)
PEP-01	-15.82	-48.88	(*69*) ForestPlots.net
PEP-02	-15.79	-48.83	(*69*) ForestPlots.net
PIM-01	-10.72	-49.45	Present study (Ricardo F. Haidar)
PIR-01	-6.16	-47.97	Present study (Ricardo F. Haidar)
PLM-01	-10.22	-48.36	Present study (Ricardo F. Haidar)
PNB-01	-15.62	-47.90	Present study (Cássia B. R. Munhoz)
PNCV	-14.14	-47.77	Present study (Sabrina Miranda)
PNT-01	-15.60	-47.66	Present study (Cássia B. R. Munhoz)
POA-02	-10.80	-51.82	(*69*) (ForestPlots.net)
PRT-01	-17.00	-46.75	Present study (Cássia B. R. Munhoz)
PTC-01	-18.78	-46.33	Present study (Cássia B. R. Munhoz)
RBM-01	-10.44	-48.81	Present study (Ricardo F. Haidar)
RDV-01	-12.75	-45.94	Present study (Cássia B. R. Munhoz)
RPT-02	-18.13	-43.36	(*69*) (ForestPlots.net)
SAT-02	-10.40	-50.64	(*69*) (ForestPlots.net)
SAV-01	-12.18	-48.01	Present study (Ricardo F. Haidar)
SD	-12.71	-45.81	Present study (Sabrina Miranda)
SDR-01	-16.02	-50.07	Present study (Sabrina Miranda)
SDT-01	-16.03	-50.07	Present study (Sabrina Miranda)
SEC-01	-19.36	-43.62	(*69*) (ForestPlots.net)
SEC-02	-19.36	-43.62	(*69*) (ForestPlots.net)
SEC-03	-19.37	-43.62	(*69*) (ForestPlots.net)
SEV-02	-17.79	-43.64	(*69*) (ForestPlots.net)
SIL	-16.64	-48.65	Present study (Sabrina Miranda)
SMT-01	-12.82	-51.77	(*69*) (ForestPlots.net)
SMT-03	-12.83	-51.77	(*69*) (ForestPlots.net)
SON-01	-10.60	-46.57	Present study (Ricardo F. Haidar)
SOR-01	-12.80	-55.98	(*69*) (ForestPlots.net)
SRM-01	-13.57	-48.23	Present study (Cássia B. R. Munhoz)
STA-01	-11.59	-48.97	Present study (Ricardo F. Haidar)
STT-01	-12.58	-48.62	Present study (Ricardo F. Haidar)
TER-01	-13.62	-46.28	(*69*) (ForestPlots.net)

TOC-01	-7.81	-47.94	Present study (Ricardo F. Haidar)
TOC-02	-13.00	-48.53	Present study (Ricardo F. Haidar)
VALE	-15.91	-47.84	Present study (Sabrina Miranda)
ZEN-CDG	-15.23	-55.54	Present study (Zenésio Finger)
ZEN-SAL	-15.94	-55.67	Present study (Zenésio Finger)

Supplementary Table 2. Hyperdominant families of the Cerrado savanna. Species richness and abundance of hyperdominant trees in the Cerrado savanna with the main species with the highest dominance within each family. The first six families together contribute more than half of all trees in the Cerrado savanna.

Family	Richness	N	Main dominant species
Vochysiaceae	21	37261	*Qualea grandiflora*
Fabaceae	96	35393	*Hymenaea stigonocarpa*
Malpighiaceae	22	12791	*Byrsonima coccolobifolia*
Myrtaceae	63	11903	*Eugenia dysenterica*
Dilleniaceae	3	11036	*Curatella americana*<br>*Davilla elliptica*<br>*Davilla grandiflora*
Ochnaceae	6	7280	*Ouratea hexasperma*
Calophyllaceae	9	7235	*Kielmeyera coriacea*
Melastomataceae	27	7072	*Miconia albicans*
Apocynaceae	18	6396	*Aspidosperma tomentosum*
Erythroxylaceae	10	5087	*Erythroxylum suberosum*

56.MAPBIOMAS v.8.0 (Brasil) https://brasil.mapbiomas.org/estatisticas/

57.Bueno, M.L. *et al.* Effects of Quaternary climatic fluctuations on the distribution of Neotropical savanna tree species. *Ecography* **40**, 403-414 (2017). doi: 10.1111/ecog.01860

58.Antonelli, A. *et al.* Geological and climatic influences on mountain biodiversity. *Nat. Geosci*. **11**, 718-725 (2018). doi: 10.1038/s41561-018-0236-z

59.Pfeilsticker, T.R. *et al.* Genetic and functional leaf trait diversity throughout the distribution of two Cerrado tree species: Testing the centre-periphery hypothesis. *J. Biogeogr*. **48**, 2258-2274 (2021). doi: 10.1111/jbi.14148

60.Pivello, V.R. The use of fire in the cerrado and Amazonian rainforests of Brazil: past and present. *Fire Ecol*. 7(1): 24-39 (2011). doi: 10.4996/fireecology.0701024

61.Silva, P.S. *et al.* Putting fire on the map of Brazilian savanna ecoregions. *J. Environ. Manage*. **296**, e113098 (2021). doi: 10.1016/j.jenvman.2021.113098

62.Almeida, S. *Cerrado: Espécies Vegetais Úteis* (EMBRAPA, 1998), p. 496.

63.Coimbra, C.E., Flowers, N.M., Salzano, F.M., Santos, R.V. *The Xavante in transition: health, ecology, and bioanthropology in Central Brazil* (University of Michigan Press, 2004). doi: 10.1525/aa.2004.106.3.614

64.Rocha, T.E.S., Silva, R.P.D., Nascimento, M.M.D. Changing dietary habits among Akwen Xerente. *Rev. Esc. Enferm. USP*. **50**, 93-97 (2016). doi: 10.1590/S0080-623420160000300014

65.Santos, M.A., Paula, M.E., Garavello, E., Reichardt, F.V. Práticas alimentares Xavante: não soberania e insegurança alimentar *Rev. Bras. Desenv. Terr. Sustent*. **6**, 28-46 (2020). doi: 10.5380/guaju.v6i2.76818

66.Ayres, M.C.C. *et al.* Químicos das folhas de *Qualea grandiflora*: Atribuição dos dados de RMN de dois flavonóides glicosilados acilados diastereoisoméricos. *Quím. Nova*. **31**, 1481-1484 (2008). doi: 10.1590/S0100-40422008000600038

67.Alvarez, F. (2024). Cerrado hyperdominance. figshare. Dataset. https://doi.org/10.6084/m9.figshare.28020971.v1
