# Supplementary material for: Tree species hyperdominance and rarity in the South American Cerrado
Source: Commun Biol. 2025 May 3;8:695. doi: 10.1038/s42003-025-07623-w (PMC12049495; doi:10.1038/s42003-025-07623-w)
Supplement: Supplementary file 2 — Reporting Summary [file 42003_2025_7623_MOESM2_ESM.pdf]

nature portfolio

Corresponding author(s): Alvarez Facundo

Last updated by author(s): Jan 21, 2025

Reporting Summary

Nature Portfolio wishes to improve the reproducibility of the work that we publish. This form provides structure for consistency and transparency in reporting. For further information on Nature Portfolio policies, see our Editorial Policies and the Editorial Policy Checklist.

Statistics

For all statistical analyses, confirm that the following items are present in the figure legend, table legend, main text, or Methods section.

n/a	Confirmed	
☐	☒	The exact sample size (*n*) for each experimental group/condition, given as a discrete number and unit of measurement
☒	☐	A statement on whether measurements were taken from distinct samples or whether the same sample was measured repeatedly
☒	☐	The statistical test(s) used AND whether they are one- or two-sided<br>*Only common tests should be described solely by name; describe more complex techniques in the Methods section.*
☒	☐	A description of all covariates tested
☒	☐	A description of any assumptions or corrections, such as tests of normality and adjustment for multiple comparisons
☒	☐	A full description of the statistical parameters including central tendency (e.g. means) or other basic estimates (e.g. regression coefficient) AND variation (e.g. standard deviation) or associated estimates of uncertainty (e.g. confidence intervals)
☒	☐	For null hypothesis testing, the test statistic (e.g. *F*, *t*, *r*) with confidence intervals, effect sizes, degrees of freedom and *P* value noted<br>*Give P values as exact values whenever suitable.*
☐	☐	For Bayesian analysis, information on the choice of priors and Markov chain Monte Carlo settings
☒	☐	For hierarchical and complex designs, identification of the appropriate level for tests and full reporting of outcomes
☒	☐	Estimates of effect sizes (e.g. Cohen's *d*, Pearson's *r*), indicating how they were calculated

Our web collection on statistics for biologists contains articles on many of the points above.

Software and code

Policy information about availability of computer code

Data collection	We used data from multiple standardized vegetation inventories conducted across various plots within the Cerrado Biome, covering approximately 2 million km². The sampling encompassed all available regions with completed inventories, including both vegetation data and relevant metadata. This approach ensured a comprehensive representation of the biome's spatial and ecological diversity, enabling robust analyses of biodiversity patterns across the Cerrado (https://doi.org/10.6084/m9.figshare.28020971.v1).
Data analysis	For the analyses, we used the R software, version 4.2.3 (https://doi.org/10.6084/m9.figshare.28020971.v1).

For manuscripts utilizing custom algorithms or software that are central to the research but not yet described in published literature, software must be made available to editors and reviewers. We strongly encourage code deposition in a community repository (e.g. GitHub). See the Nature Portfolio guidelines for submitting code & software for further information.

Data

Policy information about availability of data

All manuscripts must include a data availability statement. This statement should provide the following information, where applicable:

- Accession codes, unique identifiers, or web links for publicly available datasets
- A description of any restrictions on data availability
- For clinical datasets or third party data, please ensure that the statement adheres to our policy

All data supporting the findings of this study are available in the article and its Supplementary Information (https://doi.org/10.6084/m9.figshare.28020971.v1). The vegetation inventory data and the plot location can be accessed in Table 1 and Supplementary Table 1.

Research involving human participants, their data, or biological material

Policy information about studies with human participants or human data. See also policy information about sex, gender (identity/presentation), and sexual orientation and race, ethnicity and racism.

Reporting on sex and gender	-
Reporting on race, ethnicity, or other socially relevant groupings	-
Population characteristics	-
Recruitment	-
Ethics oversight	-

Note that full information on the approval of the study protocol must also be provided in the manuscript.

Field-specific reporting

Please select the one below that is the best fit for your research. If you are not sure, read the appropriate sections before making your selection.

☐ Life sciences ☐ Behavioural & social sciences ☒ Ecological, evolutionary & environmental sciences

For a reference copy of the document with all sections, see nature.com/documents/nr-reporting-summary-flat.pdf

Ecological, evolutionary & environmental sciences study design

All studies must disclose on these points even when the disclosure is negative.

Study description	We assessed and confirmed the existence of hyperdominance (abundance >50%) of trees (diameter of 5 cm at 30 cm above the ground) in the Cerrado biome in 222 1-hectare plots. We also estimated the number of species that the biome hosts (~1,605 tree species) and the number of trees it has lost in the last three decades (~24 billion trees).
Research sample	We utilized vegetation inventory plots located within the geographic boundaries of the Cerrado. Our study focused on self-supporting woody-stemmed plants with a minimum diameter of 5 cm at 30 cm above the ground, situated in areas not affected by floods or water table fluctuations. We incorporated 1 ha plots compiled from the literature and various laboratories and research centers, representing an unprecedented regional collaborative effort in the Cerrado. Out of a total of 764 studies reviewed, only 120 met our specific criteria, yielding a final tally of 222 plots, including those from virtual platforms.
Sampling strategy	The sample size was not selected based on statistical criteria but was instead chosen as the largest available, encompassing all surveys with inventory data on structure and/or floristic composition conducted to date for the Cerrado Biome. This effort likely represents the largest and most comprehensive sample for this biome. We excluded samples with very small area, those with non-standard procedures, or from vegetation types other than typical cerrado (e.g., wetlands, gallery forests, open grasslands, and others). In summary, we utilized the most extensive available sample for the Cerrado Biome, encompassing all regions and sampling efforts conducted to date.
Data collection	We utilized vegetation inventory plots located within the geographic boundaries of the Cerrado. Our study focused on self-supporting woody-stemmed plants with a minimum diameter of 5 cm at 30 cm above the ground, situated in areas not affected by floods or water table fluctuations. We incorporated 1 ha plots compiled from the literature and various laboratories and research centers, representing an unprecedented regional collaborative effort in the Cerrado. Out of a total of 764 studies reviewed, only 120 met our specific criteria, yielding a final tally of 222 plots, including those from virtual platforms.
Timing and spatial scale	Data were collected and updated from the 1990s to the present, within the geographic limits of the Cerrado biome (3° — 24° S; 41° — 63° W).

Data exclusions	We excluded domestic/exotic species and those classified as subshrubs or lianas.
Reproducibility	We provided a detailed description of the methodology used for biodiversity analyses and the statistical procedures. The entire process is outlined step by step to enable replication in the Cerrado Biome or any other tropical biome globally, even across different vegetation types. The procedures are thoroughly detailed in both the Materials and Methods section of the main text and in the supplementary materials (https://doi.org/10.6084/m9.figshare.28020971.v1)
Randomization	Randomization in the data collection process is neither applicable nor relevant to this study, as we utilized an existing vegetation database covering the entire biome and incorporating all available and comparable samples.
Blinding	We did not apply blinding procedures, as all data were provided by their authors for publication in this manuscript or were already published in other scientific papers.

Did the study involve field work? ☒ Yes ☐ No

Field work, collection and transport

Field conditions	Not applicable. Study conducted with data already collected.
Location	The study was conducted using vegetation data from the entire Cerrado Biome, which spans a total area of approximately two million km² in Central Brazil, between latitudes 5° and 20° S and longitudes 45° and 60° W. The Cerrado is classified as a tropical Aw biome, according to the Köppen's Classification, with two well-defined seasons: a dry season from April to September and a rainy season from October to April. The predominant vegetation is tropical savanna (cerrado sensu stricto). The primary soil type is a deep, well-drained dystrophic latosol, although other soil types are also present, ranging from deep sandy soils (neosols) to shallow concretionary soils, almost all of which are dystrophic.
Access & import/export	Not applicable. Study conducted with data already collected.
Disturbance	Not applicable. Study conducted with data already collected.

Reporting for specific materials, systems and methods

We require information from authors about some types of materials, experimental systems and methods used in many studies. Here, indicate whether each material, system or method listed is relevant to your study. If you are not sure if a list item applies to your research, read the appropriate section before selecting a response.

Materials & experimental systems

n/a	Involved in the study	
☒	☐	Antibodies
☒	☐	Eukaryotic cell lines
☒	☐	Palaeontology and archaeology
☒	☐	Animals and other organisms
☒	☐	Clinical data
☒	☐	Dual use research of concern
☐	☒	Plants

Methods

n/a	Involved in the study	
☒	☐	ChIP-seq
☒	☐	Flow cytometry
☒	☐	MRI-based neuroimaging

Plants

Seed stocks	-
Novel plant genotypes	-
Authentication	-
